# Modeling and Performance Analysis of an Improved Movement-Based Location Management Scheme for Packet-Switched Mobile Communication Systems

**DOI:** 10.1155/2014/812657

**Published:** 2014-03-09

**Authors:** Yun Won Chung, Jae Kyun Kwon, Suwon Park

**Affiliations:** ^1^School of Electronic Engineering, Soongsil University, 369 Sangdoro, Dongjak-gu, Seoul 156-743, Republic of Korea; ^2^Department of Electronic Engineering, Yeungnam University, 280 Daehak-ro Gyeongsan, Gyeongbuk 712-749, Republic of Korea; ^3^Department of Electronics and Communications Engineering, Kwangwoon University, 20 Gwangun-ro, Nowon-gu, Seoul 139-701, Republic of Korea

## Abstract

One of the key technologies to support mobility of mobile station (MS) in mobile communication systems is location management which consists of location update and paging. In this paper, an improved movement-based location management scheme with two movement thresholds is proposed, considering bursty data traffic characteristics of packet-switched (PS) services. The analytical modeling for location update and paging signaling loads of the proposed scheme is developed thoroughly and the performance of the proposed scheme is compared with that of the conventional scheme. We show that the proposed scheme outperforms the conventional scheme in terms of total signaling load with an appropriate selection of movement thresholds.

## 1. Introduction

One of the key technologies to support mobility of mobile station (MS) in mobile communication systems is location management which consists of location update and paging. In location update, an MS informs network of its current location information whenever it changes its location area (LA) [1]. Then, if there is an incoming call for an MS, the location information of the MS is retrieved and network sends paging requests to all the base stations within the retrieved LA to find the current cell of the called MS. To perform location update and paging, however, signaling load is generated and this depends on the size of LA. If the size of LA is small, an MS updates its location frequently. Then, location update signaling load is high and paging signaling load is low. On the other hand, if the size of LA is large, location update signaling load is low and paging signaling load is high. Therefore, there is a tradeoff between location update signaling load and paging signaling load, from the aspect of LA size.

In most mobile communication systems, such as GSM, GPRS, UMTS, and LTE, zone-based location update scheme is widely used, where an MS updates its location whenever it changes its current zone which is defined as a fixed group of cells. In GSM [2], LA is defined as a zone. In GPRS [1], routing area (RA) is defined as a zone for packet-switched (PS) data service and the size of RA is generally smaller than that of LA. In UMTS [1], URA (UTRAN registration area), which is smaller than RA, is defined as a zone for a more fine-grained location management of MSs. In LTE [3], tracking area (TA) is defined as a zone. For efficient location management TA list (TAL) is also defined in LTE, where there is no location update if an MS moves within TAs belonging to the same TAL assigned to the MS.

Current LA, RA, URA, and TA, however, are generally of fixed size for all MSs and they do not accommodate diverse traffic and mobility characteristics of MSs. For example, if an MS has high call-to-mobility ratio (CMR), small LA, RA, URA, and TA are more appropriate. On the other hand, if an MS has low CMR, large LA, RA, URA, and TA are more appropriate. Thus, there is no fixed size of LA, RA, URA, and TA appropriate for all MSs with diverse traffic and mobility characteristics. In order to solve this problem, dynamic location update schemes have been proposed. Distance-based [4, 5], timer-based [6, 7], and movement-based [8–13] schemes are representative examples of dynamic location update. In these schemes, the condition for location update is adaptively configured to individual MSs to accommodate different traffic and mobility characteristics of MSs. In distance-based location update scheme [4, 5], an MS updates its location whenever the distance from the last updated cell reaches a predefined distance threshold. In timer-based location update scheme [6, 7], an MS performs a location update whenever the predefined time threshold is elapsed from last updated time. Finally, in movement-based location update scheme, an MS performs a location update whenever the number of cell crossings from the last updated cell reaches a predefined movement threshold [8–13]. In this paper, movement-based location management scheme is considered, which combines the strength of both distance-based and timer-based schemes, that is, good performance and easy implementation.

Lots of works on movement-based location management scheme have been carried out [8–13]. In [8], a movement-based location update scheme with selective paging was proposed and the performance was analyzed. In movement-based location update, there is a movement counter which keeps the number of cells visited since the last location update. An MS updates its location whenever the counter reaches a predefined value, that is, movement threshold *d*, and then resets the counter value to 0. If there is an incoming call to an MS, network pages all the cells which can be reached within a movement threshold *d* movements from the last updated cell. Selective paging scheme based on a shortest-distance-first (SDF) scheme is used to reduce paging signaling load. In [10], cell-level location of MSs is predicted based on the assumption that the movement of MSs follows routine trajectories and selective polling strategy is used to reduce signaling load. In [11], the performance of movement-based location management scheme with home location register/visitor location register (HLR/VLR) architecture is thoroughly analyzed using mathematical analysis, by relaxing a simple exponential distribution assumption of cell and LA residence times. In [12], an optimal sequential paging scheme for movement-based location management was proposed using optimal movement threshold based on MSs' movement statistics. In [13], a shape of TAL is proposed based on movement-based location management. Then, the performance of the proposed TAL scheme is analyzed in LTE femtocells. In [9], the authors developed an embedded Markov chain model to investigate the performance of movement-based location management scheme, derived closed-form analytical formulas for the signaling costs, and analyzed the effects of various parameters on the signaling cost.

In PS systems, a conventional movement-based scheme with single movement threshold, however, is not appropriate, since MSs in PS systems have bursty data traffic characteristics. Since there are short idle period between data packets in a session and long idle period between data sessions [14], small and large movement thresholds may be more appropriate for a short and long idle periods, respectively. In this paper, an improved movement-based location management scheme with two movement thresholds, that is, a small movement threshold *d*_1_ for a short idle period between data packets in a session and a large movement threshold *d*_2_ for a long idle period between data sessions, is proposed. The analytical modeling for location update and paging costs of the proposed scheme is developed thoroughly and the performance of the proposed scheme is compared with those of the conventional scheme. We note that the concept of the proposed improved movement-based location management scheme was introduced in our preliminary work in [15] but it did not contain neither detailed algorithm nor performance analysis results. Also, although the idea of using multiple movement thresholds is similar to using multiple timer thresholds in our previous work for an improved timer-based location management [7], this work provides significant extensions from that in [7]. Firstly, a new location update criterion of movement-based location management scheme is considered, instead of simple timer-based location update criterion, and a detailed algorithm for the proposed scheme is presented. Also, a new analytical modeling considering location update criterion of movement-based location management is developed thoroughly. Finally, the effect of cell residence time variance on signaling load is newly analyzed.

The remainder of this paper is organized as follows.  Section 2 proposes an improved movement-based location management scheme. The performance of the proposed scheme is analyzed in Section 3 by developing an analytical methodology. In Section 4 numerical examples are provided. Conclusion and future work are presented in Section 5.

## 2. An Improved Movement-Based Location Management Scheme

In PS systems, MS states are managed in networks for efficient use of radio resources and signaling networks. In GPRS,* ready* and* standby* states are defined. In* ready* sate, an MS is more likely to receive or transmit data packets in short time and updates its location for every cell change. If a session between an MS and a network is completed and there is no packet exchange until the expiration of* ready timer*, it is more likely that the MS does not send or receive a new data packet for some time and the MS moves to the* standby* state, where RA-based location information is managed. The* ready* timer is reset and restarted when there is a packet exchange between an MS and a network. In UMTS,* cell-connected*,* URA-connected*, and* PMM-idle* states are defined, and cell-, URA-, and RA-based location information is managed, respectively.* Inactivity* timer and* periodic URA update* timer are used to control MS state transitions. In LTE, cell- and TA-based location information is managed in* active* and* idle* states.

In this paper, two MS states are defined based on the traffic characteristics of MSs, as in GPRS and LTE, and these two state are denoted as* ready* and* standby*, for notational convenience. Two movement thresholds *d*_1_ and *d*_2_ (*d*_1_ ≤ *d*_2_) are proposed for* ready* and* standby*, respectively. However, the analytical methodology developed in this paper can be extended to any PS systems which have more MS states. In this paper, state transitions between MS states are controlled by inactivity counter which counts the number of cells crossed since the last location update. An inactivity counter is reset whenever there is data packet exchange between MS and network. If there is no data packet exchange between MS and network until the inactivity counter is expired, an MS moves from* ready* state to* standby* state. We note that the signaling packet exchange does not reset the inactivity counter.

**Figure 1:**
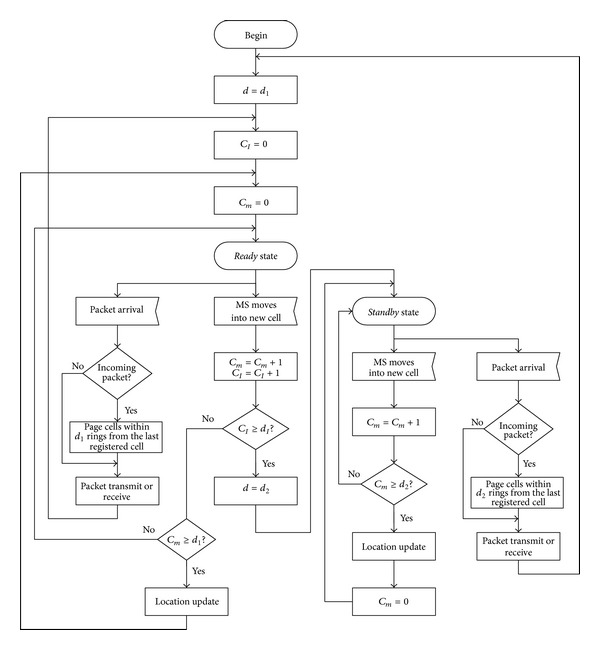
Flow chart for an improved movement-based location management scheme.

Figure 1 illustrates the flowchart for the proposed scheme. If an MS attaches to the network, the sate of the MS is set at* ready* state and the movement threshold *d* is initialized to *d*_1_. The value of an inactivity counter *C*_*I*_ and the value of movement counter *C*_*m*_ are initialized to 0. Inactivity counter is used to control the transition between* ready* and* standby* states. Movement counter is used to track the number of cells crossed since the last location update. The MS in the* ready* state moves into one of two cases according to the next events. If the MS moves into a new cell, the values of movement counter and inactivity counter are increased by 1 (*C*_*m*_ = *C*_*m*_ + 1 and *C*_*I*_ = *C*_*I*_ + 1) and the inactivity counter value is compared with the value of inactivity movement threshold, *d*_*I*_. If *C*_*I*_ ≥ *d*_*I*_, movement threshold *d* is changed to *d*_2_ and the state is changed from* ready* state to* standby* state. Otherwise, a movement counter value is compared with *d*_1_. If *C*_*m*_ ≥ *d*_1_, location update is performed and movement counter value is reset to 0. If *C*_*m*_ < *d*_1_, there is no location update and the MS just returns back to the* ready* state and waits for the next event. If there is any packet arrival, it is processed appropriately according to whether it is an incoming or an outgoing packet. If it is an incoming packet, network pages cells within *d*_1_ rings of cells from the last registered cell. Otherwise, an outgoing packet is transmitted. After receiving or transmitting packets, the values of movement counter and inactivity counter are initialized to 0.

In* standby* state, the MS moves into one of two cases according to the next events. If the MS moves into a new cell, the value of movement counter is increased by 1 (*C*_*m*_ = *C*_*m*_ + 1) and the movement counter value is compared with the value of movement threshold in the* standby* state, *d*_2_. If *C*_*m*_ ≥ *d*_2_, location update is performed and it returns back to* standby* state. The value of movement counter is reset to 0. Otherwise, the MS just returns back to* standby* state and waits for the next event. If there is any packet arrival, it is processed appropriately according to whether it is an incoming or an outgoing packet. If it is an incoming packet, networks page cells within *d*_2_ rings of cells from the last registered cell. Otherwise, an outgoing packet is transmitted. After receiving or transmitting packets, movement threshold is changed to *d*_1_, the values of movement counter and inactivity counter are initialized to 0, and the MS state changes to* ready* state.

## 3. Performance Analysis of the Proposed Scheme

In this section, we develop an analytical methodology to investigate the performance of the proposed scheme. Firstly, we describe mobility and traffic models considered in this paper. Based on these models, the number of location updates and the number of paged cells are derived. Finally, total signaling load is obtained as a weighted sum of the number of location updates and the number of paged cells.

### 3.1. Mobility and Traffic Models

**Figure 2:**
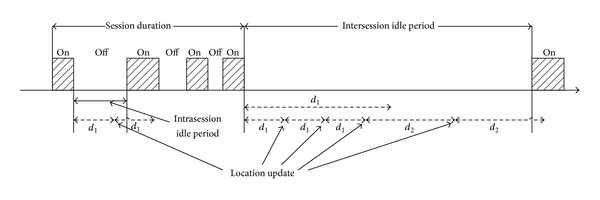
Timing diagram for the proposed movement-based scheme and data traffic modeling.

Figure 2 shows a timing diagram for the proposed movement-based scheme with data traffic modeling based on ETSI packet model with an ON/OFF source, which is adopted from [14]. A session duration consists of alternating ON/OFF periods, where a burst of data packets is generated during ON period and no packet is transmitted during OFF period [14]. The *d*_1_, *d*_2_, and *d*_*I*_ are movement threshold in* ready* state, movement threshold in* standby* state, and inactivity movement threshold, respectively. If the value of movement counter is larger than inactivity movement threshold, the movement threshold value is changed to *d*_2_. There are two idle periods, that is, intersession idle period *t*_*p*1_ and intrasession idle period *t*_*p*2_. It is assumed that the intersession idle period *t*_*p*1_ has a gamma distribution with mean 1/*λ*_*p*1_ and variance *V*_*p*1_ [14]. The gamma distribution with mean 1/*λ*_*p*1_ = *η*/*λ* and variance *V*_*p*1_ = *η*/*λ* ^2^ has the following probability density function:

(1) $$\begin{array}{c} f_{t_{p1}}\left(t\right)=\frac{\lambda e^{-\lambda t}{\left(\lambda t\right)}^{\eta -1}}{\Gamma \left(\eta \right)}, \end{array}$$

where Γ(*η*) = ∫_*z*=0_^*∞*^ *z* ^*η*−1^ *e* ^−*z*^ *dz* is the gamma function, and *η* and *λ* denote the shape parameter and the scale parameter, respectively. The intrasession idle period *t*_*p*2_, that is, OFF period, is assumed to follow a Pareto distribution with mean 1/*λ*_*p*2_ and infinite variance, which is widely used to model actual packet data traffic [14]. The Pareto distribution has the following density function:

(2) $$\begin{array}{c} f_{t_{p2}}\left(t\right)=\{\begin{array}{cc} \left(\frac{\beta }{l}\right){\left(\frac{l}{t}\right)}^{\beta +1} & \text{if}\;t\geq l\\ 0 & \text{if}\;t<l, \end{array} \end{array}$$

where *β* describes the heaviness of the tail of the distribution and *l* is the minimum value that the distribution can have. The expectation of the Pareto distribution is *E*[*t*_*p*2_] = (*βl*/(*β* − 1)). The number of OFF periods in a communication session is assumed to follow a geometric distribution with mean *α*/(1 − *α*)  (0 ≤ *α* < 1) based on the ETSI data traffic model [14].

In conventional movement-based scheme, only one movement threshold value *d*_0_ is generally used. In the proposed scheme, however, two movement threshold values are assumed. An MS initially updates its location for every *d*_1_ unit of movement during idle period. Inactivity counter *d*_*I*_ starts at the beginning of the idle period and it is reset whenever there is a data packet exchange. It is assumed that the interarrival time of packets during ON period is very short and there is no inactivity counter expiration during ON period. If there is no data packet transmission during the inactivity counter, the inactivity counter expires and a new movement threshold *d*_2_ is used for location update. If both *d*_1_ and *d*_*I*_ expire at the same time, we assume that only the expiration of *d*_*I*_ is valid and a new threshold *d*_2_ is used for location update, that is, the next location update occurs (*d*_2_ − *d*_1_) movements later.

Cell residence time *T*_*C*_*i*__ at the *i*th cell is assumed to have an independently and identically distributed Erlang distribution with mean 1/*λ*_*m*_ = *m*/*λ*_*c*_ and variance *V*_*m*_ = *m*/*λ*_*c*_^2^, and the PDF is given by

(3) $$\begin{array}{c} f_{T_{C_{i}}}\left(t\right)=\frac{\lambda_{c}^{m}t^{m-1}}{\left(m-1\right)!}e^{-\lambda_{c}t}\;\text{for}\;\;t\geq 0, \end{array}$$

where *m* = 1,2, 3,…. Erlang distribution is selected because it can be easily extended into hyper-Erlang distribution, which has been proven as a good approximation to many other distributions as well as measured data [16]. In order to derive the number of movements, we use the property that the sum of *m* random variables with exponential distribution with mean 1/*λ*_*c*_ follows an Erlang distribution with mean 1/*λ*_*m*_ = *m*/*λ*_*c*_ and variance *V*_*m*_ = *m*/*λ*_*c*_^2^. Thus, an Erlang distribution consists of *m* Poisson arrivals with rate *λ*_*c*_. Thus, the probability that the number of movements during time *t* is *i* is given by

(4) $$\begin{array}{c} P_{N_{\text{move}}}\left(i;t\right)=\overset{mi+m-1}{\underset{j=mi}{\sum }}P_{N_{p}}\left(j;t\right)‍, \end{array}$$

where *P*_*N*_*p*__(*j*; *t*) is the probability that the number of Poisson arrivals during time *t* is *j* and is expressed by

(5) $$\begin{array}{c} P_{N_{p}}\left(j;t\right)=\frac{{\left(\lambda_{c}t\right)}^{j}e^{-\lambda_{c}t}}{j!}. \end{array}$$

### 3.2. Derivation of the Number of Location Updates

For conventional scheme, the number of location updates during time *t* is derived as

(6) Nuconv(t)=∑i=0∞⌊id0⌋PNmove(i;t)=∑i=0∞⌊id0⌋∑j=mimi+m−1(λct)je−λctj!.

The total numbers of location updates during *t*_*p*1_ and *t*_*p*2_ of conventional scheme are calculated as

(7) Nu1conv=∫0∞Nuconv(t)ftp1(t)dt=∫0∞∑i=0∞⌊id0⌋∑j=mimi+m−1(λct)je−λctj!λe−λt(λt)η−1Γ(η)dt=∑i=0∞⌊id0⌋∑j=mimi+m−1∫0∞(λct)je−λctj!λe−λt(λt)η−1Γ(η)dt=∑i=0∞⌊id0⌋∑j=mimi+m−11j!·Γ(η) ×∫0∞(λct)je−λctλe−λt(λt)η−1dt=∑i=0∞⌊id0⌋∑j=mimi+m−11j!·Γ(η)(λc)j(λ)η(λc+λ)j+η ×∫0∞((λc+λ)t)j+η−1e−(λc+λ)td((λc+λ)t)=∑i=0∞⌊id0⌋∑j=mimi+m−1(λc)j(λ)η(λc+λ)j+ηΓ(j+η)j!·Γ(η)=∑i=0∞⌊id0⌋∑j=mimi+m−1(λc)j(λ)η(λc+λ)j+ηΓ(j+η)Γ(j+1)Γ(η)=∑i=0∞⌊id0⌋∑j=mimi+m−1(λc)j(λ)η(λc+λ)j+ηΓ(j+η)jΓ(j)Γ(η)=∑i=0∞⌊id0⌋∑j=mimi+m−1(λc)j(λ)η(λc+λ)j+η1j·B(j,η)=(λλc+λ)η∑i=0∞⌊id0⌋∑j=mimi+m−1(λcλc+λ)j1j·B(j,η),

where *B*(*j*, *η*) is the beta function,

(8) Nu2conv=∫0∞Nuconv(t)ftp2(t)dt=∫l∞∑i=0∞⌊id0⌋∑j=mimi+m−1(λct)je−λctj!(βl)(lt)β+1dt=∑i=0∞⌊id0⌋∑j=mimi+m−1∫l∞(λct)je−λctj!(βl)(lt)β+1dt=∑i=0∞⌊id0⌋∑j=mimi+m−1β·(λcl)βj! ×∫l∞(λct)j−β−1e−λctd(λct)=∑i=0∞⌊id0⌋∑j=mimi+m−1β·(λcl)βj!Γ(j−β,λcl)=β·(λcl)β∑i=0∞⌊id0⌋∑j=mimi+m−1Γ(j−β,λcl)j!,

where Γ(*j* − *β*, *λ*_*c*_ *l*) is the incomplete gamma function.

For proposed scheme, the number of location updates during time *t* is derived as

(9) Nuprop(t)=∑i=0d∗⌊id1⌋PNmove(i;t) +∑i=d∗+1∞(d∗d1+⌊i−d∗d2⌋)PNmove(i;t)=∑i=0d∗⌊id1⌋∑j=mimi+m−1(λct)je−λctj! +∑i=d∗+1∞(d∗d1+⌊i−d∗d2⌋)∑j=mimi+m−1(λct)je−λctj!,

where d* is defined as d* = (⌈*d*_*I*_/*d*_1_⌉ − 1)*d*_1_. The total numbers of location updates during *t*_*p*1_ and *t*_*p*2_ of the proposed scheme are calculated as

(10) Nu1prop=∫0∞Nuprop(t)ftp1(t)dt=∫0∞∑i=0d∗⌊id1⌋∑j=mimi+m−1(λct)je−λctj!λe−λt(λt)η−1Γ(η)dt +∫0∞∑i=d∗+1∞(d∗d1+⌊i−d∗d2⌋) ×∑j=mimi+m−1(λct)je−λctj!λe−λt(λt)η−1Γ(η)dt=∑i=0d∗⌊id1⌋∑j=mimi+m−1∫0∞(λct)je−λctj!λe−λt(λt)η−1Γ(η)dt +∑i=d∗+1∞(d∗d1+⌊i−d∗d2⌋) ×∑j=mimi+m−1∫0∞(λct)je−λctj!λe−λt(λt)η−1Γ(η)dt=(λλc+λ)η ×∑i=0d∗{⌊id1⌋∑j=mimi+m−1(λcλc+λ)j1j·B(j,η)} +(λλc+λ)η ×∑i=d∗+1∞{(d∗d1+⌊i−d∗d2⌋)×∑j=mimi+m−1(λcλc+λ)j1j·B(j,η)},

(11) Nu2prop=∫0∞Nuprop(t)ftp2(t)dt=∫l∞∑i=0d∗⌊id1⌋∑j=mimi+m−1(λct)je−λctj!(βl)(lt)β+1dt +∫l∞∑i=d∗+1∞(d∗d1+⌊i−d∗d2⌋) ×∑j=mimi+m−1(λct)je−λctj!(βl)(lt)β+1dt=∑i=0d∗⌊id1⌋∑j=mimi+m−1∫l∞(λct)je−λctj!(βl)(lt)β+1dt +∑i=d∗+1∞(d∗d1+⌊i−d∗d2⌋) ×∑j=mimi+m−1∫l∞(λct)je−λctj!(βl)(lt)β+1dt=β(λcl)β[∑i=0d∗{⌊id1⌋∑j=mimi+m−1Γ(j−β,λcl)j!}+∑i=d∗+1∞{(d∗d1+⌊i−d∗d2⌋)×∑j=mimi+m−1Γ(j−β,λcl)j!}].

### 3.3. Derivation of the Number of Paged Cells

Paging is performed at the beginning of each ON period. It is assumed that no paging is needed at the packet intervals during the ON period because the intervals are too short and thus, the location of an MS can be tracked at cell level implicitly by data packet transmission. The number of paged cells depends on the movement threshold value used when the ON period begins. In this paper, hexagonal cell is assumed for mathematical tractability. We use a selective paging scheme [8] to find the current cell of the called MS, where network pages the called MS starting from the cell where the MS updated its location lastly and outwards, in a shortest distance first order until the called MS is found [8].

For conventional scheme, the number of cells paged during time *t* is derived as

(12) Nvconv(t)=∑j=0 ∞∑k=0j%d0Nc(k)δ(k,j%d0)PNmove(j;t)=∑j=0 ∞∑k=0j%d0(1+3k+3k2)δ(k,j%d0) ×∑i=mjmj+m−1(λct)ie−λcti!,

where *N*_*c*_(*k*) is the number of cells from the center cell to the *k*th ring and *δ*(*k*, *j*%*d*_0_) is the probability that after *j*%*d*_0_ movements, the distance between the current and the center cells is *k* [8], where % is modular operation.

The total numbers of cells paged during *t*_*p*1_ and *t*_*p*2_ of conventional scheme are calculated as

(13) Nv1conv=∫0∞Nvconv(t)ftp1(t)dt=∫0∞∑j=0  ∞∑k=0j%d0(1+3k+3k2)δ(k,j%d0) ×∑i=mjmj+m−1(λct)ie−λcti!λe−λt(λt)η−1Γ(η)dt=(λλc+λ)η∑j=0  ∞∑k=0j%d0(1+3k+3k2)δ(k,j%d0) ×∑i=mjmj+m−1(λcλc+λ)i1i·B(i,η),

(14) Nv2conv=∫0∞Nvconv(t)ftp2(t)dt=∫l∞∑j=0  ∞∑k=0j%d0(1+3k+3k2)δ(k,j%d0) ×∑i=mjmj+m−1(λct)ie−λcti!(βl)(lt)β+1dt=∑j=0  ∞∑k=0j%d0(1+3k+3k2)δ(k,j%d0) ×∑i=mjmj+m−1∫l∞(λct)ie−λcti!(βl)(lt)β+1dt=β·(λcl)β∑j=0  ∞∑k=0j%d0(1+3k+3k2)δ(k,j%d0) ×∑i=mjmj+m−1Γ(i−β,λcl)i!.

For proposed scheme, the number of cells paged during time *t* is derived as

(15) Nvprop(t)=∑j=0 d∗−1∑k=0j%d1Nc(k)δ(k,j%d1)PNmove(j;t) +∑j=d∗∞∑k=0(j−d∗)%d2Nc(k)δ(k,(j−d∗)%d2) ×PNmove(j;t)=∑j=0  d∗−1∑k=0j%d1(1+3k+3k2)δ(k,j%d1) ×∑i=mjmj+m−1(λct)ie−λcti! +∑j=d∗∞∑k=0(j−d∗)%d2(1+3k+3k2)δ(k,(j−d∗)%d2) ×∑i=mjmj+m−1(λct)ie−λcti!.

The total numbers of cells paged during *t*_*p*1_ and *t*_*p*2_ of proposed scheme are calculated as

(16) Nv1prop=∫0∞Nvprop(t)ftp1(t)dt=∫0∞∑j=0  d∗−1∑k=0j%d1(1+3k+3k2)δ(k,j%d1) ×∑i=mjmj+m−1(λct)ie−λcti!λe−λt(λt)η−1Γ(η)dt +∫0∞∑j=d∗∞∑k=0(j−d∗)%d2(1+3k+3k2)×δ(k,(j−d∗)%d2) ×∑i=mjmj+m−1(λct)ie−λcti!λe−λt(λt)η−1Γ(η)dt=∑j=0  d∗−1∑k=0j%d1(1+3k+3k2)δ(k,j%d1) ×∑i=mjmj+m−1∫0∞(λct)ie−λcti!λe−λt(λt)η−1Γ(η)dt +∑j=d∗∞∑k=0(j−d∗)%d2(1+3k+3k2) ×δ(k,(j−d∗)%d2) ×∑i=mjmj+m−1∫0∞(λct)ie−λcti!λe−λt(λt)η−1Γ(η)dt=(λλc+λ)η ×{∑j=0d∗−1{∑k=0j%d1(1+3k+3k2)δ(k,j%d1)×∑i=mjmj+m−1(λcλc+λ)i1i·B(i,η)}+∑j=d∗∞{∑k=0(j−d∗)%d2(1+3k+3k2)‍×δ(k,(j−d∗)%d2)×∑i=mjmj+m−1(λcλc+λ)i×1i·B(i,η)}},

(17) Nv2prop=∫0∞Nvprop(t)ftp2(t)dt=∫l∞∑j=0   d∗−1∑k=0j%d1(1+3k+3k2)δ(k,j%d1)×∑i=mjmj+m−1(λct)ie−λcti!(βl)(lt)β+1dt +∫l∞∑j=d∗∞∑k=0(j−d∗)%d2(1+3k+3k2)×δ(k,(j−d∗)%d2) ×∑i=mjmj+m−1(λct)ie−λcti!(βl)(lt)β+1dt=∑j=0   d∗−1∑k=0j%d1(1+3k+3k2)δ(k,j%d1) ×∑i=mjmj+m−1∫l∞(λct)ie−λcti!(βl)(lt)β+1dt +∑j=d∗∞∑k=0(j−d∗)%d2(1+3k+3k2) ×δ(k,(j−d∗)%d2) ×∑i=mjmj+m−1∫l∞(λct)ie−λcti!(βl)(lt)β+1dt=β·(λcl)β{∑j=0d∗−1{∑k=0j%d1(1+3k+3k2)×δ(k,j%d1)×∑i=mjmj+m−1Γ(i−β,λcl)i!}+∑j=d∗∞{∑k=0(j−d∗)%d2(1+3k+3k2)×δ(k,(j−d∗)%d2)×∑i=mjmj+m−1Γ(i−β,λcl)i!}}=β·(λcl)β{∑j=0d∗−1{∑i=mjmj+m−1Γ(i−β,λcl)i!×∑k=0j%d1(1+3k+3k2)×δ(k,j%d1)}+∑j=d∗∞{∑i=mjmj+m−1Γ(i−β,λcl)i!×∑k=0(j−d∗)%d2(1+3k+3k2)×δ(k,(j−d∗)%d2)}}.

### 3.4. Signaling Load

For signaling load analysis, it is assumed that the weight for performing a location update is *U* and the weight for paging at one cell is *V*. Location update signaling load and paging signaling load of the conventional scheme during a cycle of consecutive communication session and intersession idle period are obtained based on the geometric distribution of the number of OFF periods in a communication session [14] as follows:

(18) $$\begin{array}{c} U_{\text{conv}}=U\left(N_{u_{1}}^{\text{conv}}+\frac{\alpha }{1-\alpha }N_{u_{2}}^{\text{conv}}\right), \end{array}$$

(19) $$\begin{array}{c} P_{\text{conv}}=V\left(N_{v_{1}}^{\text{conv}}+\frac{\alpha }{1-\alpha }N_{v_{2}}^{\text{conv}}\right). \end{array}$$

From (18) and (19), the total signaling load for location update and paging of the conventional scheme is

(20) $$\begin{array}{c} T_{\text{conv}}=U_{\text{conv}}+P_{\text{conv}}. \end{array}$$

Likewise, location update signaling load and paging signaling load of the proposed scheme during a cycle of consecutive communication session and intersession idle period are obtained as follows:

(21) $$\begin{array}{c} U_{\text{prop}}=U\left(N_{u_{1}}^{\text{prop}}+\frac{\alpha }{1-\alpha }N_{u_{2}}^{\text{prop}}\right), \end{array}$$

(22) $$\begin{array}{c} P_{\text{prop}}=V\left(N_{v_{1}}^{\text{prop}}+\frac{\alpha }{1-\alpha }N_{v_{2}}^{\text{prop}}\right). \end{array}$$

From (21) and (22), the total signaling load for location update and paging of the conventional scheme is

(23) $$\begin{array}{c} T_{\text{prop}}=U_{\text{prop}}+P_{\text{prop}}. \end{array}$$

## 4. Numerical Examples

### 4.1. Signaling Load Comparison

**Figure 3:**
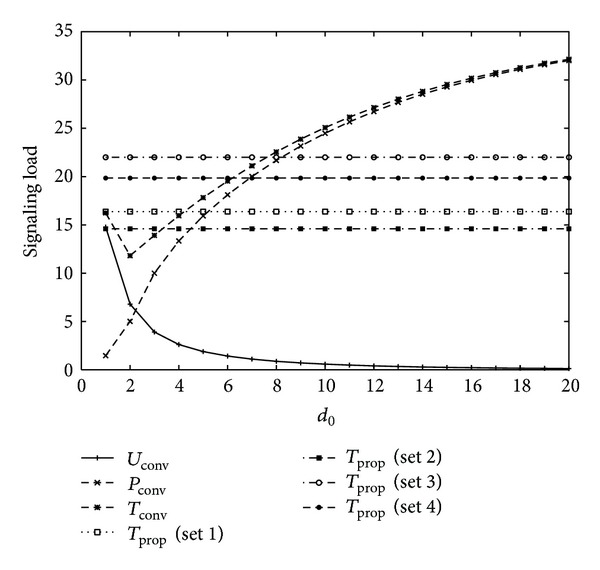
Signaling load for 1/*λ*_*c*_ = 10.5∗20/3600(*h*) (low mobility), 1/*λ* = 10.5∗100/3600(*h*) (short intersession idle period).

In numerical examples, we compare the signaling load of the conventional and proposed schemes for varying mobility and intersession idle period.  Figure 3 shows signaling load for varying the value of movement threshold in conventional scheme, that is, *d*_0_, with four different sets of movement threshold values, as shown in Table 1 for *U* = 2, *V* = 1, *η* = 1, *b* = 1.2, *m* = 1, *α* = 0.8, 1/*λ*_*c*_ = 10.5∗20/3600(*h*) (low mobility), 1/*λ* = 10.5∗100/3600(*h*) (short intersession idle period), and *l* = (*b* − 1)/*b*∗*λ*_*c*_. The location update signaling load of the conventional scheme decreases as *d*_0_ increases. On the other hand, paging signaling load of the conventional scheme increases as *d*_0_ increases. Thus, the total signaling load of the conventional scheme follows a convex shape with optimal movement threshold *d*_0_ = 2. Since the total signaling load of the proposed scheme does not depend on *d*_0_, it has a constant value. The proposed schemes with Sets 1 and 2, that is, smaller values of *d*_1_ and *d*_2_, have less signaling load than those with Sets 3 and 4, that is, higher values of *d*_1_ and *d*_2_. The proposed schemes with Sets 1 and 2 have smaller signaling load than conventional scheme for most values of *d*_0_. Also, the proposed schemes with Sets 3 and 4 have smaller signaling load than conventional scheme if the value of *d*_0_ is large.

**Figure 4:**
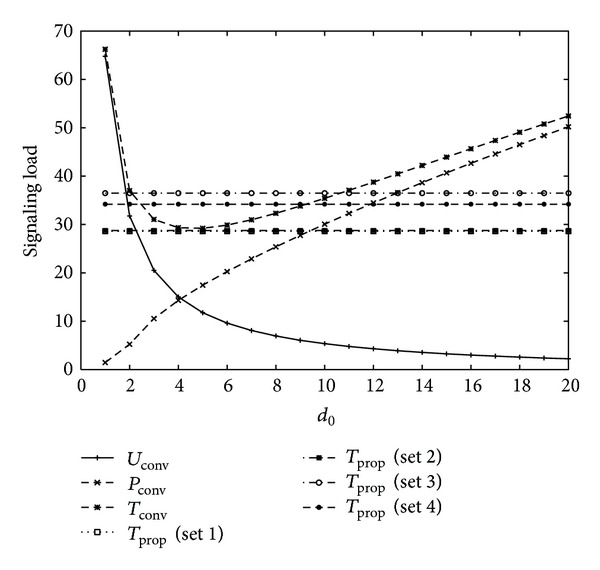
Signaling load for 1/*λ*_*c*_ = 10.5∗20/3600(*h*) (low mobility), 1/*λ* = 10.5∗600/3600(*h*) (long inter-session idle period).

Figure 4 shows signaling load for varying the value of *d*_0_ with four different sets of movement threshold values, as shown in Table 1 for *U* = 2, *V* = 1, *η* = 1, *b* = 1.2, *m* = 1, *α* = 0.8, 1/*λ*_*c*_ = 10.5∗20/3600(*h*) (low mobility), 1/*λ* = 10.5∗600/3600(*h*) (long intersession idle period), and *l* = (*b* − 1)/*b*∗*λ*_*c*_. The total signaling load of the conventional scheme follows a convex shape with optimal movement threshold *d*_0_ = 5. Similar to Figure 3, the proposed schemes with Sets 1 and 2 have less signaling load than those with Sets 3 and 4. The proposed schemes with Sets 1 and 2 have smaller signaling load than conventional scheme for all values of *d*_0_. Also, the proposed schemes with Sets 3 and 4 have smaller signaling load than conventional scheme if the value of *d*_0_ is very small or large.

**Figure 5:**
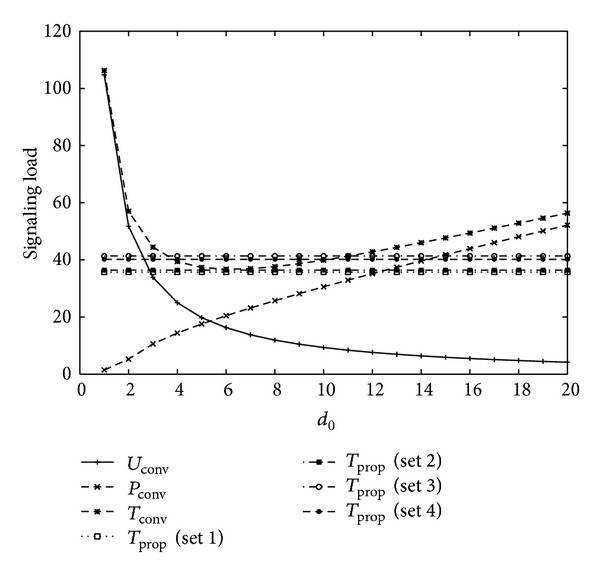
Signaling load for 1/*λ*_*c*_ = 10.5∗2/3600(*h*) (high mobility), 1/*λ* = 10.5∗100/3600(*h*) (short intersession idle period).

Figure 5 shows signaling load for varying the value of *d*_0_ with four different sets of movement threshold values, as shown in Table 1 for *U* = 2, *V* = 1, *η* = 1, *b* = 1.2, *α* = 0.8, 1/*λ*_*c*_ = 10.5∗2/3600(*h*) (high mobility), 1/*λ* = 10.5∗100/3600(*h*) (short intersession idle period), and *l* = (*b* − 1)/*b*∗*λ*_*c*_, *m* = 1. The total signaling load of the conventional scheme follows a convex shape with optimal movement threshold *d*_0_ = 6. Similar to Figures 3 and 4, the proposed schemes with Sets 1 and 2 have less signaling load than those with Sets 3 and 4. Similar to Figure 4, the proposed schemes with Sets 1 and 2 have smaller signaling load than conventional scheme for all values of *d*_0_. Also, the proposed schemes with Sets 3 and 4 have smaller signaling load than conventional scheme if the value of *d*_0_ is very small or large.

**Figure 6:**
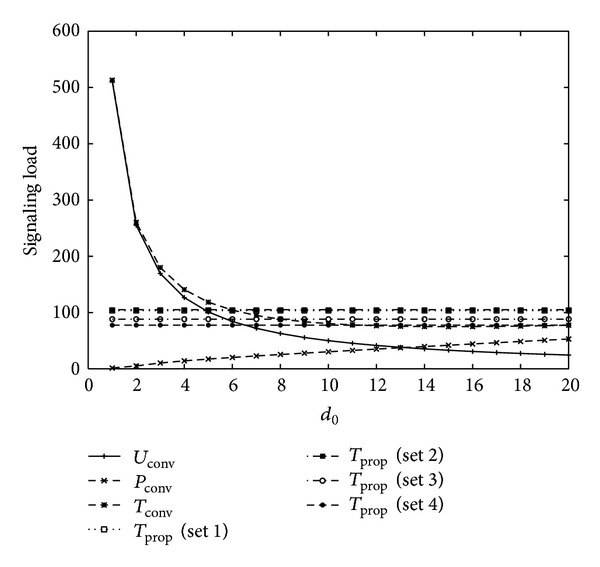
Signaling load for 1/*λ*_*c*_ = 10.5∗2/3600(*h*) (high mobility), 1/*λ* = 10.5∗600/3600(*h*) (long intersession idle period).

Figure 6 shows signaling load for varying the value of *d*_0_ with four different sets of movement threshold values, as shown in Table 1 for *U* = 2, *V* = 1, *η* = 1, *b* = 1.2, *α* = 0.8, 1/*λ*_*c*_ = 10.5∗2/3600(*h*) (high mobility), 1/*λ* = 10.5∗600/3600(*h*) (long intersession idle period), and *l* = (*b* − 1)/*b*∗*λ*_*c*_, *m* = 1. The total signaling load of the conventional scheme follows a convex shape with optimal movement threshold *d*_0_ = 11. Contrary to Figures 3, 4, and 5, the proposed schemes with Sets 1 and 2 have more signaling load than those with Sets 3 and 4. The proposed scheme with Set 4 has smaller signaling load than conventional scheme for all values of *d*_0_. Also, the proposed schemes with Sets 1, 2, and 3 have smaller signaling load than conventional scheme if the value of *d*_0_ is very small.

From the results of Figures 3, 4, 5, and 6, it is concluded that the optimal movement threshold value of the conventional scheme varies for different combinations of mobility and traffic environments of an MS, and the signaling load of the conventional scheme rapidly increases if movement threshold value is far from the optimal movement threshold value. On the other hand, the proposed scheme with Sets 1 and 2 performs better than the conventional scheme for most combinations of mobility and traffic environments of an MS with stable performance, except for high mobility and high intersession idle period. For high mobility and high intersession idle period, the proposed scheme with Set 4 performs better than the conventional scheme. Therefore, the proposed scheme outperforms the conventional scheme with an appropriate selection of movement threshold values.

### 4.2. Effect of Cell Residence Time Variance on Signaling Load

**Figure 7:**
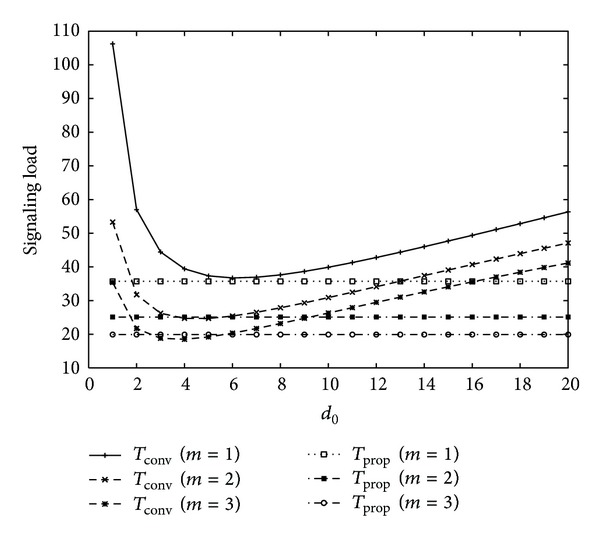
Effect of cell residence time variance on signaling load (1/*λ*_*c*_ = 10.5∗2/3600(*h*) (high mobility), 1/*λ* = 10.5∗100/3600(*h*) (short intersession idle period)).

Figure 7 shows signaling load for varying the value of movement threshold in conventional scheme with three different sets of *m* values in order to show the effect of variance on cell residence time, for *U* = 2, *V* = 1, *η* = 1, *b* = 1.2, 1/*λ*_*c*_ = 10.5∗2/3600(*h*) (high mobility), and 1/*λ* = 10.5∗100/3600(*h*) (short intersession idle period), *l* = (*b* − 1)/*b*∗*λ*_*c*_, and *α* = 0.8 with Set 1 in Table 1. As can be seen in Figure 7, signaling load in both the conventional and proposed schemes decreases as the values of *m* increases, that is, the variance increases. That is, it is shown that higher variance on cell residence time results in less signaling load. Also, for small values of *m*, the signaling load of the proposed scheme is always smaller than that of the conventional scheme. It is concluded that the proposed scheme works well irrespective of cell residence time variance.

## 5. Conclusion and Future Work

In this paper, an improved movement-based location management scheme with two movement thresholds is proposed to accommodate bursty data traffic characteristics of PS services. The analytical modeling for location update and paging signaling loads of the proposed scheme was developed thoroughly and the performance of the proposed scheme was compared with that of the conventional scheme in detail, for varying the mobility and traffic characteristics of MSs. We show that the proposed scheme outperforms the conventional scheme with an appropriate selection of movement thresholds, irrespective of cell residence time variance. In our future work, we will develop an adaptive selection of an appropriate set of movement thresholds depending on mobility and traffic characteristics of an MS, based on the analysis results of this paper.

## Figures and Tables

**Table 1:** Sets of movement threshold values.

	*d*_1_	*d*_2_	*d*_*I*_
Set 1	3	6	6
Set 2	3	6	12
Set 3	6	12	12
Set 4	6	12	24
